# Two subphenotypes of septic acute kidney injury are associated with different 90-day mortality and renal recovery

**DOI:** 10.1186/s13054-020-02866-x

**Published:** 2020-04-15

**Authors:** Renske Wiersema, Sakari Jukarainen, Suvi T. Vaara, Meri Poukkanen, Päivi Lakkisto, Hector Wong, Adam Linder, Iwan C. C. van der Horst, Ville Pettilä

**Affiliations:** 1 Department of Critical Care, University of Groningen, University Medical Center Groningen, Groningen, The Netherlands; 2 Division of Intensive Care Medicine, Department of Anesthesiology, Intensive Care and Pain Medicine, University of Helsinki and Helsinki University Hospital, Helsinki, Finland; 3 Department of Anesthesiology and Intensive Care, Lapland Central Hospital, Rovaniemi, Finland; 4 Department of Clinical Chemistry and Hematology, University of Helsinki and Helsinki University Hospital and Minerva Foundation Institute for Medical Research, Helsinki, Finland; 5 Division of Critical Care Medicine, Department of Pediatrics, Cincinnati Children’s Hospital Medical Center, University of Cincinnati College of Medicine, Cincinnati, OH USA; 6 Department of Clinical Sciences, Division of Infection Medicine, Lund University, Lund, Sweden; 7 Department of Intensive Care, Maastricht University Medical Centre+, University Maastricht, Maastricht, The Netherlands

**Keywords:** Subphenotypes, Acute kidney injury, Sepsis, LCA, Renal recovery, Mortality

## Abstract

**Background:**

The pathophysiology of septic acute kidney injury is inadequately understood. Recently, subphenotypes for sepsis and AKI have been derived. The objective of this study was to assess whether a combination of comorbidities, baseline clinical data, and biomarkers could classify meaningful subphenotypes in septic AKI with different outcomes.

**Methods:**

We performed a post hoc analysis of the prospective Finnish Acute Kidney Injury (FINNAKI) study cohort. We included patients admitted with sepsis and acute kidney injury during the first 48 h from admission to intensive care (according to Kidney Disease Improving Global Outcome criteria). Primary outcomes were 90-day mortality and renal recovery on day 5. We performed latent class analysis using 30 variables obtained on admission to classify subphenotypes. Second, we used logistic regression to assess the association of derived subphenotypes with 90-day mortality and renal recovery on day 5.

**Results:**

In total, 301 patients with septic acute kidney injury were included. Based on the latent class analysis, a two-class model was chosen. Subphenotype 1 was assigned to 133 patients (44%) and subphenotype 2 to 168 patients (56%). Increased levels of inflammatory and endothelial injury markers characterized subphenotype 2. At 90 days, 29% of patients in subphenotype 1 and 41% of patients in subphenotype 2 had died. Subphenotype 2 was associated with a lower probability of short-term renal recovery and increased 90-day mortality.

**Conclusions:**

In this post hoc analysis, we identified two subphenotypes of septic acute kidney injury with different clinical outcomes. Future studies are warranted to validate the suggested subphenotypes of septic acute kidney injury.

## Background

Acute kidney injury (AKI) has been an important research focus within intensive care medicine in recent years [1]. Incidence, risk factors, and outcome of AKI have been widely described [2–4]. The treatment options for AKI are limited, and consequently, long-term morbidity and mortality are substantial [5, 6]. The lack of effective treatment options is partly explained by AKI being a complex and multifactorial syndrome [3] and, additionally, currently inadequately understood underlying pathophysiological mechanisms [7–9].

Septic AKI accounts for approximately 50% of AKI cases in critically ill patients [7]. Given the heterogeneity of critically ill patients and their underlying illnesses, it is plausible that several subphenotypes of AKI exist, analogous to those in acute respiratory distress syndrome (ARDS) [10, 11] and in sepsis [12]. Recently, Bhatraju et al. described two possible subphenotypes of septic AKI in critically ill patients which had different outcomes in terms of renal recovery and mortality [13]. Identification of diagnostic subphenotypes of septic AKI may be crucial in order to improve prognostication and to identify different patient groups responding differently to treatment [14], as previously observed in AKI subphenotypes regarding vasopressin [13]. Schaub et al. suggested to pursue this type of research and investigate whether other markers that were not included by Bhatraju et al. could also aid in identifying subphenotypes of (septic) AKI [15].

Heterogeneity in the development, evolution, treatment effects, and outcomes of septic AKI may be explained by genetic factors, different comorbidities, other organ dysfunction, and expression of biomarkers following underlying pathophysiological processes [16]. These factors are not included in the current AKI KDIGO classification, using only serum creatinine and urinary output [17], which may be seen as an important limitation [18].

Multiple variables, including comorbidities, baseline clinical data, and biomarkers, are of potential use in identifying subphenotypes. Multicentre studies have aimed to identify biomarkers that could predict development, evolution, and outcome of AKI with mostly disappointing results [19–21]. We have previously evaluated multiple possible biomarkers of AKI in the FINNAKI cohort [2, 22–30].

We hypothesized that a combination of comorbidities, baseline clinical data, and multiple biomarkers could aid in the identification of subphenotypes in septic AKI upon ICU admission [28, 31]. Accordingly, we performed a post hoc analysis in critically ill patients with septic AKI using the prospectively collected FINNAKI cohort data [2] aiming at identifying subphenotypes of septic AKI.

## Methods

### Study population

Of the 2901 critically ill patients included in the FINNAKI study [2], we included all patients with septic AKI with biomarker data. The FINNAKI study was a prospective, observational, multicentre study in which 17 Finnish ICUs participated between 1 September 2011 and 1 February 2012. For the present study, we excluded 617 of the 918 septic FINNAKI patients [32]: 160 patients who did not give consent for biomarker analysis, 404 patients without AKI during the first 48 h of admission, and 53 patients with more than 6 missing values (50%) for the 12 biomarkers (E-Fig. 3).

The Ethics Committee of the Department of Surgery in Helsinki University Central Hospital approved the FINNAKI study protocol with written informed consent from patients or their next of kin and the use of deferred consent. This study was reported in adherence to STROBE (Additional file 2).

### Definitions

We defined AKI as any AKI of KDIGO stage one and higher within the first 48 h of admission, using the complete KDIGO criteria based on serum creatinine, urinary output, the combination of creatinine and urinary output, and use of renal replacement therapy (RRT) [33]. Sepsis was defined according to the initial ACCP/SCCM Consensus Conference Committee definition and assessed prospectively by the researchers [34]. Modified SOFA on ICU admission was calculated as the SOFA score but leaving out the central nervous system (CNS) component, and the renal component was determined solely based on creatinine levels since urinary output data were not available on admission. Average vasopressor load (μg/kg/min) during the first 24 h was calculated from norepinephrine equivalent vasopressors administered: [norepinephrine equivalents (μg/kg/min)] = [norepinephrine μg/kg/min)] + [dopamine (μg/kg/min)]/2 + [epinephrine (μg/kg/min)].The primary outcome was 90-day mortality. The second outcome was renal recovery on day 5, as renal recovery has been shown to be associated with improved outcomes after AKI and to allow comparison with similar studies. Day 5 was the last day of clinical data collection. Renal recovery on day 5 was defined as survival to 5 days and no AKI, which was based on the full KDIGO criteria using both serum creatinine and urinary output on day 5.

### Variables

We included previously analysed biomarkers, previously chosen based on the literature due to their association with the evolution or outcomes of AKI [35]. Clinical variables for the latent class analysis (LCA) were selected by clinical judgement and previous studies [36]. We restricted all class-defining variables to either permanent patient characteristics or variables measured from 24 h prior to admission to 2 h after admission. E-Table 3 lists all chosen class-defining variables.

### Statistical analysis

We present data as means (with standard deviations (SD)) or medians (with interquartile ranges (IQR)) depending on distributions. Student’s *t* test, Mann-Whitney *U* test, and Pearson’s chi-squared test were used as appropriate. Outcomes were calculated as odds ratios (OR) with 95% confidence intervals (CI). A *p* value of < 0.05 was considered statistically significant. *p* values were not corrected for multiple comparisons in the analyses. Analyses were performed using Stata 15 and R (version 3.6.0).

First, we performed LCA to identify subphenotypes in septic AKI. Due to missing data, we performed a multiple imputation procedure on the data for LCA. Data were multiply imputed 31 times, using data for 615 septic patients, of which 301 constitute the analysed population of septic AKI patients. The 314 septic patients without AKI in the first 48 h were only included in the imputation model to improve the imputation procedure. We performed the LCA model for the 301 septic AKI patients on each imputed dataset separately, and the final class assignment was determined by taking the majority votes of the 31 models for each patient (see Additional file 1 for details). The optimal number of latent classes was decided by considering the Bayesian information criterion (BIC), the number of classes, class sizes, and entropy (see Additional file 1 for details).

Second, we used logistic regression to assess whether class membership was associated with different 90-day mortality and renal recovery on day 5. We controlled for age and sex and measures of disease severity: APACHE II, admission modified SOFA score, KDIGO AKI stage, and presence of chronic or acute liver failure.

Last, as a confirmatory analysis, we performed a sensitivity analysis using data 24 h prior to and after ICU admission.

## Results

Of 2901 FINNAKI study patients, 354 fulfilled inclusion criteria of which 301 patients had adequate biomarker data. Most of the characteristics of the included and excluded patients were similar (E-Table 4). Of the 301 patients, 166 patients (55%) had AKI either on admission or within 24 h and 135 patients (45%) were diagnosed with AKI between 24 and 48 h. Of all patients, 127 patients (42%) had AKI based on creatinine only, 51 on urine output only (17%), and 123 patients (41%) on both or the use of RRT. Two- and three-class models were explored, and the two-class model for the LCA was selected based on BIC, entropy, and class sizes (see Additional file 1 for details including a comparison to the three-class model). In total, 133 patients (43%) had phenotype 1 and 168 patients (57%) had phenotype 2. The mean probability of class membership in the model was 0.960 for subphenotype 1 and 0.959 for subphenotype 2.

**Fig. 1:**
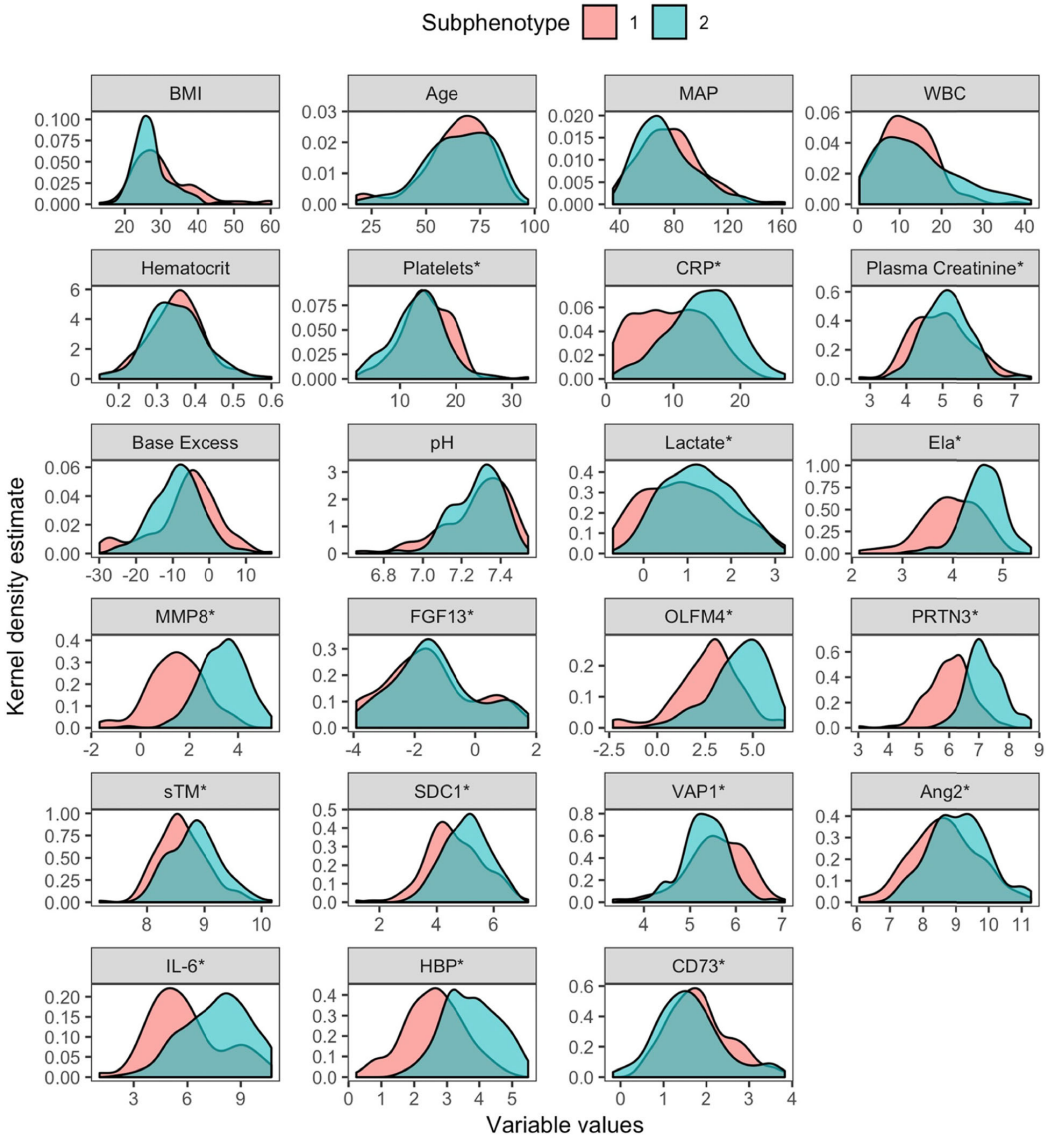
Combined graphs of variable distribution in different sub-phenotypes. Description: variables names with an asterisk were plotted as either natural log or square root transformed. BMI, body mass index; MAP, mean arterial pressure; WBC, white blood cell count; CRP, C-reactive protein; Ela, neutrophil elastase 2; MMP8, matrix metalloproteinase 8; FGF13, fibroblast growth factor 13; OLFM4, olfactomedin 4; PRTN3, proteinase 3; sTM, soluble thrombomodulin; SDC1, syndecan-1; VAP1, vascular adhesion protein 1; Ang2, angiotensin 2; IL-6, interleukin-6; HBP, heparin-binding protein

**Fig. 2:**
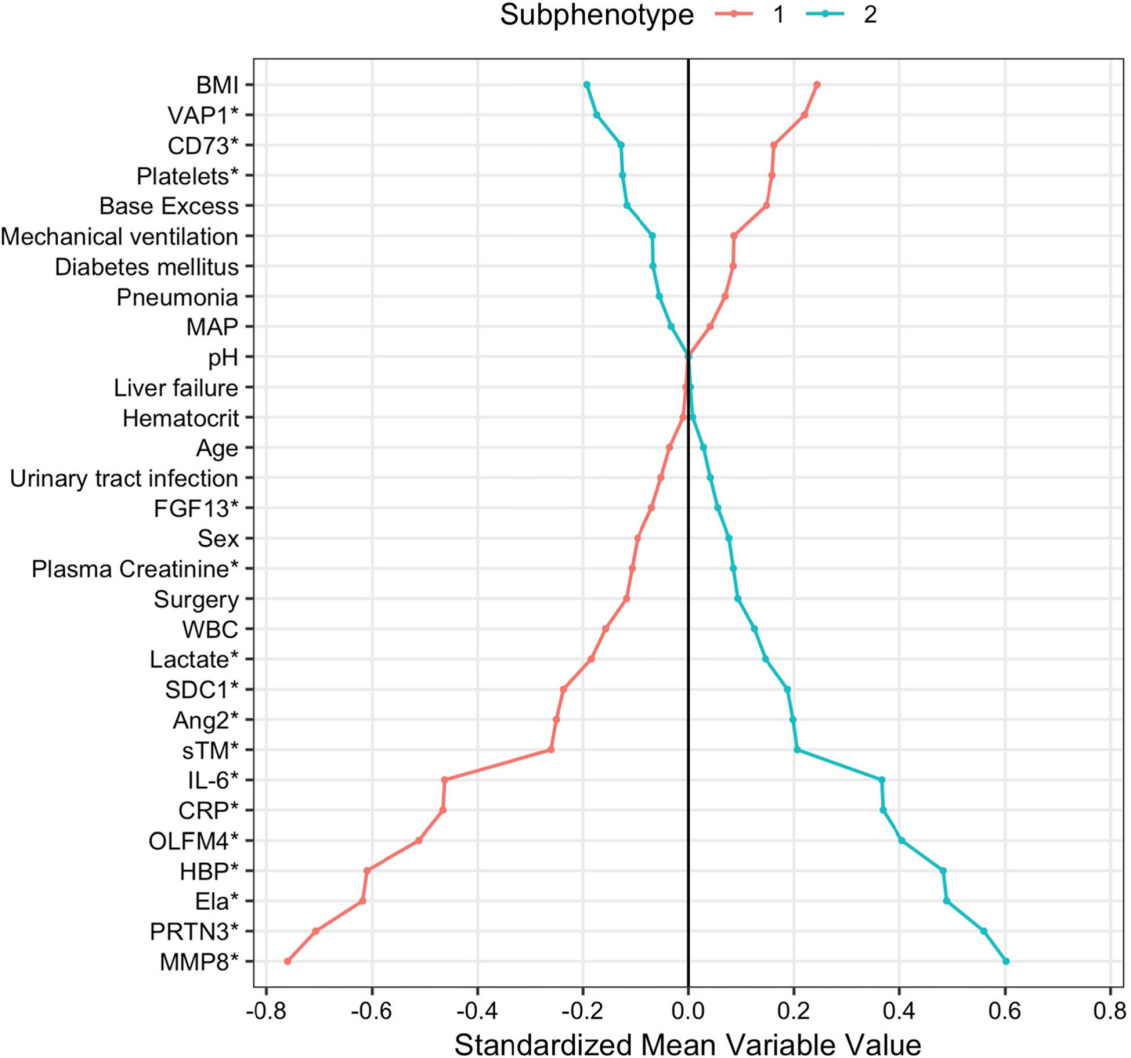
Comparison of class-defining variables by class. Description: variables names with an asterisk were plotted as either natural log or square root transformed. Every variable was standardized to a mean of 0 and SD of 1. Group means of standardized values are shown by class. The distance between the lines corresponds to the standardized mean difference between groups. BMI, body mass index; MAP, mean arterial pressure; WBC, white blood cell count; CRP, C-reactive protein; Ela, neutrophil elastase 2; MMP8, matrix metalloproteinase 8; FGF13, fibroblast growth factor 13; OLFM4, olfactomedin 4; PRTN3, proteinase 3; sTM, soluble thrombomodulin; SDC1, syndecan-1; VAP1, vascular adhesion protein 1; Ang2, angiotensin 2; IL-6, interleukin-6; HBP, heparin-binding protein

Baseline characteristics of all 301 patients are shown in Table 1. Variable distribution differed across the subphenotypes (Fig. 1). Compared to subphenotype 1, subphenotype 2 was characterized by higher levels of heparin-binding protein (HBP), neutrophil elastase 2 (Ela), proteinase 3 (PRTN3), olfactomedin-4 (OLFM4), and matrix metalloproteinase 8 (MMP8). Figure 2 shows the standardized class-defining variable values by class, ranked by standardized mean difference (SMD). Of the clinical variables, patients with subphenotype 2 had lower BMI (27.3 vs 30.3 kg/m^2^, *p* < 0.001), were more likely to receive vasopressors (64% vs 48%, *p* = 0.007), and had a lower prevalence of chronic kidney disease (4% vs 13%, *p* = 0.005). The timing of AKI diagnosis did not differ (*p* = 0.94). The median vasopressor load was higher in patients with subphenotype 2 (0.28 vs 0.18 μg/kg/min, *p* = 0.009) as was the median fluid balance at 72 h (8286 vs 6738 mL, *p* = 0.012) (Table 1). In the sensitivity analysis using 24-h data, 130 patients (43%) had subphenotype 1 and 171 patients (57%) had subphenotype 2 (E-Table 5), and distinctive variables and outcomes were the same as in the primary model (E-Fig. 4).

**Table 1:** Characteristics of study population per subphenotype

	Subphenotype 1 (*n* = 133)	Subphenotype 2 (*n* = 168)	*p* value
Age, years (SD)	64 (15)	65 (15)	0.58
Sex, male	88 (66%)	97 (58%)	0.14
BMI, kg/m^2^ (SD)	30.3 (8.5)	27.3 (5.1)	< 0.001
Operative admission	28 (21.2%)	51 (30.4%)	0.074
Diabetes mellitus	44 (33.1%)	44 (26.2%)	0.19
Chronic liver failure	7 (5.3%)	3 (1.8%)	0.098
Chronic kidney disease	17 (13.1%)	7 (4.2%)	0.005
Baseline creatinine, mmol/L (SD)*	91.1 (55.9)	86.7 (50.3)	0.55
Admission origin			0.49
Operation room/recovery	28 (21.1%)	48 (28.6%)	
Emergency room	55 (41.4%)	60 (35.7%)	
Ward	39 (29.3%)	48 (28.6%)	
Other ICU/high-dependency unit/other	11 (8.3%)	12 (7.1%)	
Timing of AKI			0.94
Within 24 h	73 (54.9%)	93 (55.4%)	
Within 48 h	60 (45.1%)	75 (44.6%)	
Clinical variables on admission			
Urinary tract infection	12 (9.0%)	20 (11.9%)	0.42
Pneumonia	26 (19.5%)	25 (14.9%)	0.28
Mechanical ventilation	72 (54.1%)	78 (46.4%)	0.18
Vasopressors, any	64 (48.1%)	107 (63.7%)	0.007
KDIGO AKI stage			0.37
Stage 1	63 (47.4%)	67 (39.9%)	
Stage 2	22 (16.5%)	26 (15.5%)	
Stage 3	35 (26.3%)	60 (35.7%)	
Modified SOFA on admission (SD)	6.0 (2.6)	6.4 (2.5)	0.20
APACHE II score (SD)	27.3 (9.4)	27.8 (8.6)	0.66
Mean arterial pressure, mmHg (SD)	75 (21)	74 (22)	0.58
Biochemical variables on admission			
Leukocyte count, × 10^9^/L (SD)	12.1 (6.4)	14.3 (9.6)	0.027
Platelet count, × 10^9^/L (SD)	232 (125)	202 (143)	0.064
Haematocrit (SD)	0.35 (0.07)	0.35 (0.08)	0.90
CRP, nmol/L (SD)	124 (111)	234 (143)	< 0.001
pH (SD)	7.3 (0.2)	7.3 (0.1)	0.78
Highest lactate, mmol/L (SD)	4.1 (4.5)	4.5 (3.6)	0.47
Base excess, lowest (SD)	− 6.2 (9.1)	− 8.3 (6.7)	0.039
Creatinine, μmol/L (SD)	213 (230)	212 (181)	0.97
Treatment			
Fluid balance at 72 h, mL (IQR)	6738 (1868–10,169)	8286 (4202–13,671)	0.012
Vasopressor load, μg/kg/min (IQR)	0.18 (0.04–0.44)	0.28 (0.12–0.69)	0.009
Renal replacement therapy	31 (23.3%)	66 (39.3%)	0.003
Outcomes			
SOFA score on day 2 (SD)	6.3 (3.8)	8.3 (4.4)	< 0.001
SOFA score on day 3 (SD)	6.1 (3.7)	7.3 (4.3)	0.025
Renal recovery on day 5	85 (63.9%)	78 (46.4%)	0.003
In-hospital mortality	25 (18.8%)	60 (35.7%)	0.001
90-day mortality	39 (29.3%)	68 (40.5%)	0.045

Description: Data are presented as numbers (percentages) or mean (SD)/median (IQR). Vasopressors included here are norepinephrine, dobutamine, and epinephrine

*BMI* body mass index, *KDIGO* Kidney Disease Improving Global Outcome, *AKI* acute kidney injury, *SOFA* Sequential Organ Failure Assessment, *APACHE* Acute Physiology, Age, Chronic Health Evaluation, *CRP* C-reactive protein

*Baseline serum creatinine was available for 69% of patients and, if missing, estimated using the MDRD formula

### Outcomes

Renal recovery on day 5 occurred in 163 patients (54%). At day 5, 42 patients (14%) and at 90 days, 107 patients (36%) had died. Logistic regression showed that membership in subphenotype 2 was associated with both decreased short-term renal recovery and increased mortality (Table 2).

**Table 2:** Logistic regression, associations between subphenotype 2 membership and outcomes

	Unadjusted OR (95% CI)	*p* value	Adjusted* OR (95% CI)	*p* value
Renal recovery on day 5	0.49 (0.31–0.78)	0.003	0.47 (0.27–0.79)	0.005
90-day mortality	1.64 (1.01–2.67)	0.045	1.83 (1.05–3.24)	0.035

Description: ORs are for subphenotype 2 vs. subphenotype 1

*Adjusted for age, sex, liver failure, modified SOFA score, APACHE II, and KDIGO AKI stage

## Discussion

In this post hoc analysis of the multicentre prospective FINNAKI study, we identified two distinct subphenotypes of septic AKI, using variables on ICU admission in a cohort of critically ill patients. These findings remained unchanged in a model using variables obtained in the first 24 h. Importantly, subphenotype 2 was associated with decreased short-term renal recovery and increased 90-day mortality.

Of the 30 included variables used in the LCA, four biomarkers showed the greatest difference between the two subphenotypes: HBP, Ela, PRTN3, and MMP8. Increased understanding of the underlying pathophysiologic mechanisms behind the subphenotypes represented by these biomarkers could aid in the design of future trials and further help treat septic AKI. HBP is a neutrophil-derived mediator for inflammation and vascular permeability and has been reported to improve the prediction of a clinical risk model in septic patients [28]. HBP reflects vascular permeability and was higher in patients with subphenotype 2, who also received more fluids. The three other biomarkers Ela, PRTN3, and MMP8 are also neutrophil-derived proteases, which have previously been shown to accurately estimate the risk of AKI in septic children [37]. Notably, none of these four biomarkers is measured in standard clinical practice. The fact that these biomarkers appear higher in subphenotype 2 suggests that subphenotype 1 may represent a less severe form of septic AKI. Supporting this hypothesis, sCD73, a protective anti-inflammatory mediator, was higher in patients with subphenotype 1 [27]. Moreover, despite comparable SOFA and APACHE II scores on admission, this is reflected by higher vasopressor load and higher fluid balance later during admission in patients with subphenotype 2. Clinically, patients with subphenotype 1 had a higher BMI and less severe metabolic acidosis. Although the number of patients with diagnosed chronic kidney disease was higher in subphenotype 1, the baseline serum creatinine was comparable.

All previous studies except one [36] have classified AKI into different types of AKI based on renal outcome, but these subphenotypes were determined using outcome data retrospectively and not linked to distinguishable pathophysiological pathways [38, 39]. Other studies have focused on subphenotypes of AKI in very specific contexts, such as kidney transplants [40] or experimental models [41]. Recently, however, two major studies using similar methods in critically ill patients with sepsis have been published [36, 42]. Different subphenotypes of sepsis have been identified in 16,552 critically ill patients. Although this study [42] did not focus on AKI specifically, the methodology is comparable to our study using a combination of baseline and clinical data to identify subphenotypes of a heterogeneous clinical syndrome [42].

More recently, Bhatraju et al. showed that two subphenotypes of AKI could be identified based on 29 variables using comparable methods [36]. The authors constructed a simplified three-variable model based on the ratio between plasma angiopoietin 2 (Ang-2) and angiopoietin 1 (Ang-1) and s TNF receptor 1, which was able to distinguish between the subphenotypes. The model performed well. We analysed similar outcomes, primarily 90-day mortality and short-term renal recovery, although the latter is inevitably subject to competing risk of death. However, some major methodological differences exist. First, we focused on patients with septic AKI only whereas in the cohorts of Bhatraju et al., sepsis incidences range from 46% to 84% throughout the subphenotypes across the cohorts. Second, despite a large cohort of concomitant ICU patients, we were able to include only 301 patients with septic AKI in this study. Although we had to exclude 53 patients due to inadequate biomarker data, we consider the included patients to represent the cohort since their baseline characteristics were comparable to those excluded. Third, the model by Bhatraju et al. was investigated in a development cohort and thereafter validated in separate cohorts, which adds validity to their model. Fourth, however, instead of pre-empt restricted time points for biomarkers, Bhatraju et al. included biomarkers collected at various points within 24 h of AKI diagnosis, which may vary, and some were measured at study enrolment (which was, for example, 12 h after meeting criteria for inclusion in the VASST trial [43]). Of note, in our primary model, we only included data available on ICU admission, as this approach is most clinically relevant for future implementation and potential guide for treatment. Finally, we included 12 markers that reflect either endothelial dysfunction or inflammation, where a panel of inflammatory markers was most prominent, compared to Bhatraju et al. who included eight biomarkers reflecting similar underlying pathophysiology [36]. In comparison, we included 18 of those 29 variables in the Bhatraju model. Among these were two biomarkers, namely Ang-2 and interleukin-6, and both showed comparable associations with the more severe subphenotype of septic AKI, although Ang-2 was less distinctive in our results.

### Implications and generalizability

We were able to identify two subphenotypes of septic AKI that have different clinical outcomes. As our model is based on variables available on ICU admission only, we consider our observations important for potential implementation in selecting patients for trials and potentially for future therapeutic options. Our findings need further external validation in other cohorts but encourage for future (prospective) research to combine clinical and biomarker data in search for ways to decrease heterogeneity and aim for precision care [44]. If confirmed, our findings regarding additional discriminative value support the use of a biomarker panel including HBP and neutrophil-derived proteases in critically ill patients with septic AKI. Notably, the classification of the two distinct subphenotypes remained stable using 24-h clinical data increasing its clinical potential on ICU admission. Moreover, future studies could perform similar analyses in septic patients in general to assess whether a combination of biomarkers could aid in identifying patients at increased risk of adverse outcomes.

### Limitations

There are some important limitations to consider. First, there are multiple potential ways of classifying patients through unsupervised learning [45]. Even in the context of LCA, classification is dependent on the choice of class-defining variables, number of classes, and model parameters. Although we selected variables based on current literature and clinical expertise, classifying patients using some other combination of clinical data, biomarkers, and other types of data could lead to a different classification. Also, our sample size was somewhat limited for unsupervised learning. However, we mitigated this problem by first defining the final LCA model without examining the outcomes and examined associations between class membership and outcomes only after the final model was specified, thus avoiding overfitting our classification to the outcomes. Due to sample size limitations, we did not attempt to internally validate the results on a held-out dataset. Moreover, similar analyses on larger samples might end up at more than two distinct classes. Nonetheless, the lack of validation is an important limitation to our study. Second, we defined our population of patients with AKI by selecting those that had AKI within the first 48 h of admission. Although more than half had AKI during the first day, the definition of AKI still has a delay as opposed to the actual renal injury and we, therefore, consider that it did not influence our result as confirmed by the 24-h model. Moreover, we have previously shown that the majority of patients develop AKI within the first 2 days. We suspect that biomarkers measured on ICU admission would not be associated with AKI occurring later. Thus, we believe that the results are representative. Additionally, the outcome renal recovery was determined on day 5, while 7 days would be the optimal follow-up period. However, day 5 was the last observation day within this study. Third, this was a post hoc analysis of prospectively gathered clinical and laboratory data—all laboratory measurements were not available for all patients but were multiply imputed. Thus, to assess the usefulness of the subphenotypes, they would ultimately have to be validated in a prospective setting. Moreover, our sample size limited the number of classes that can be reliably studied. In a larger sample, it could be possible to determine more than two classes and assess their association with the outcomes. Yet, a previous study using a similar methodology with a larger sample size ended up with two classes as well [36]. However, we focused on septic AKI due to possible different pathophysiological mechanisms underlying different types of AKI. Fourth, sepsis was defined according to the definition by the American College of Chest Physicians/Society of Critical Care Medicine (ACCP/SCCM) [34]. This was, at the time of patient inclusion for the FINNAKI study, the used definition. Using the sepsis-3 definition was not possible given the data. Similarly, at the time, colloids were still regularly administered. It is important to note that prominent changes in process of care or definitions of disease over time may impact the clinical outcomes of sub-phenotypes. Finally, as this was an observational study with usual care, causal inferences regarding any suggested treatments cannot be drawn. Ultimately the goal will be to prospectively evaluate how different phenotypes respond to different types of treatment in randomized clinical trials stratified according to these or other detected subphenotypes of AKI.

## Conclusions

In this post hoc analysis using data from a prospective observational study, we were able to identify two subphenotypes of septic AKI with statistically significantly different 90-day mortality and rate of short-term renal recovery. The subphenotypes were primarily classified using variables on admission only but were robust to a sensitivity analysis using clinical variables of the first 24 h. These detected subphenotypes warrant prospective external validation.

## Supplementary information

**Additional file 1.** Detailed description of statistical analysis. E-Table 1: Comparison of latent class analysis models with different numbers of classes. E-Table 2: Pearson correlations of class defining variables with absolute correlations over 0.5. E-Table 3: List of variables included in multiple imputation and percentages of missing data. E-Table 4: All included variables for clustering. E-Table 5. Comparison of baseline characteristics between included and excluded patients. E-Table 6. Baseline of patients in class of 24-hour variable model. E-Table 7. Comparison of admission and 24-hour model classification. E-Figure 1: First two principal components of the variables used in the LCA, two class and three class model comparison. E-Figure 2: Heatmap of class assignments for the admission model across 31 imputations. E-Figure 3: Flowchart of patient inclusion. E-Figure 4: Standardized mean difference (SMD) plot of class defining variables of 24-hour model.

**Additional file 2.** STROBE checklist and corresponding page numbers.

## Data Availability

The datasets used and/or analysed during the current study are available from the corresponding author on reasonable request.

## Abbreviations

AKI: Acute kidney injury
ANG: Angiotensin
APACHE: Acute Physiology, Age, Chronic Health Evaluation
ARDS: Acute respiratory distress syndrome
BIC: Bayesian information criterion
Ela: Neutrophil elastase 2
HBP: Heparin-binding protein
ICU: Intensive care unit
IQR: Interquartile range
KDIGO: Kidney Disease Improving Global Outcome
LCA: Latent class analysis
MMP 8: Matrix metalloproteinase 8
OLFM4: Olfactomedin 4
OR: Odds ratio
PRTN3: Proteinase 3
RRT: Renal replacement therapy
SD: Standard deviation
SMD: Standardized mean difference
SOFA: Sequential Organ Failure Assessment
TNF: Tumour necrosis factor
