# Supplementary material for: Two subphenotypes of septic acute kidney injury are associated with different 90-day mortality and renal recovery
Source: Crit Care. 2020 Apr 15;24:150. doi: 10.1186/s13054-020-02866-x (PMC7161019; doi:10.1186/s13054-020-02866-x)
Supplement: Supplementary file 1 — Additional file 1. Detailed description of statistical analysis. E-Table 1: Comparison of latent class analysis models with different numbers of classes. E-Table 2: Pearson correlations of class defining variables with absolute correlations over 0.5. E-Table 3: List of variables included in multiple imputation and percentages of missing data. E-Table 4: All included variables for clustering. E-Table 5. Comparison of baseline characteristics between included and excluded patients. E-Table 6. Baseline of patients in class of 24-hour variable model. E-Table 7. Comparison of admission and 24-hour model classification. E-Figure 1: First two principal components of the variables used in the LCA, two class and three class model comparison. E-Figure 2: Heatmap of class assignments for the admission model across 31 imputations. E-Figure 3: Flowchart of patient inclusion. E-Figure 4: Standardized mean difference (SMD) plot of class defining variables of 24-hour model. [file 13054_2020_2866_MOESM1_ESM.docx]

**Additional file 1**

**Two subphenotypes of septic acute kidney injury are associated with 90-day mortality and renal recovery**

Authors: Renske Wiersema^1^, Sakari Jukarainen^2^, Suvi Vaara^2^, Meri Poukkanen^3^, Päivi Lakkisto^4^, Hector Wong^5^, Adam Linder^6^, Iwan C.C. van der Horst^1, 7^, Ville Pettilä^2^.

**Detailed description of statistical analysis**

**Variable transformations**

For the LCA, plotting, and logistic regression, we transformed some of the highly skewed variables. We applied a natural log transformation to all of the biomarkers, creatinine levels, and lactate. We applied a square root transformation to CRP levels, platelet count, and vasopressor load.

**Multiple imputation**

Missing data for the class defining variables were assumed to be missing at random (MAR) conditional on the observed data on variables included in the imputation model. Missing data were imputed with multiple imputation using chained equations (MICE) [1] using “mice” package version 3.5.0 [2]. Variables were selected for use in the imputation model if they were expected to either: 1) correlate with variables with missing values, or 2) correlate with the probability of missingness. Variables used in multiple imputation are presented in ESM-table 3. For each variable imputation was done using information from all other variables with a minimum spearman correlation of 0.1 and minimum proportion of usable cases of 0.4.

For imputation, we used data for 615 septic patients. These 615 patients had identical exclusion and inclusion criteria to the analyzed 301 septic AKI patients, except patients without AKI in the first 48 hours were included here. We included non-AKI patients for the imputation in order to improve the imputations. Thirty-one imputed datasets were created with 20 iterations. Convergence was monitored through plotting the means and standard deviations of imputed variables in each different imputation sequence set against the iteration number. All variables were imputed using predictive mean matching, since it is robust, general purpose hot-deck method.

Multiple imputation was used instead of using full-information maximum likelihood LCA that tolerates missing values, since multiple imputation allows us to plot the class defining variables more correctly than using complete cases only, assuming the data are not missing completely at random (MCAR).

**Latent class analysis**

We used latent class analysis (LCA) [3] (or more specifically, multivariate mixture estimation with Gaussian and categorical components) to derive latent class memberships. LCA assumes that there exists an unobserved latent class for each patient, which explains variability in the class defining variables. Or conversely, class defining variables are interpreted to be imperfect indicators of the latent class membership. Using expectation maximization, the algorithm fits mixtures of multivariate Gaussian and categorical distributions that fits the data, and outputs the assigned class memberships and probabilities of belonging in each class for each patient. LCA was performed using “MixAll” package version 1.4.2 [4], using the models “gaussian_pk_sjk” (diagonal Gaussian mixture) for continuous variables, and “categorical_pk_pjk” for categorical variables (probabilities between variables free). Continuous variables were not standardized prior to modeling, since the used model is not sensitive to the relative variances of the class-defining variables. The class defining variables were largely uncorrelated with each other. Please see ESM-table 2 for the correlations between class defining variables that had at least one correlation of above 0.5 in magnitude.

The LCA model for the 301 septic AKI patients was run on each of the 31 imputed datasets separately, and the final class assignment was determined by taking the majority votes of the 31 models for each patient. Consistency of class assignments across the variability introduced by multiple imputation was assessed by plotting a heatmap of the class assignments for each patient across imputations and was deemed adequate (ESM-figure 2).

We performed the LCA with 2, 3, and 4 classes. The number of classes was chosen to be two, based on considering the Bayesian Information Criterion (BIC), class sizes, entropy, and statistical power to examine differences in outcomes between classes (ESM-table 1). Although a 3-class model had the lowest BIC, a 2 class model was preferred primarily due to parsimony and more favorable class sizes for examining associations with outcomes. The main difference between the two class and three class models lies in class 2 (two class model) being split into two classes (ESM-figure 1). Also, 34 individuals change from class 1 (two class model) to class 2 (three class model).

**ESM-table 1. Comparison of latent class analysis models with different numbers of classes**

| No. classes | BIC | Entropy | Log likelihood | *n*, class 1 | *n*, class 2 | *n*, class 3 | *n*, class 4 |
| --- | --- | --- | --- | --- | --- | --- | --- |
| 2 | 27652.22 | 0.992 | -13520.78 | 133 | 168 |  |  |
| 3 | 27607.22 | 0.949 | -13344.19 | 137 | 106 | 58 |  |
| 4 | 27611.56 | 0.963 | -13192.26 | 117 | 70 | 62 | 52 |

* Description: Model statistics are from single LCA models run on one representative imputed dataset.

**ESM-figure 1.** First two principal components of the variables used in the LCA, two class and three class model comparison.


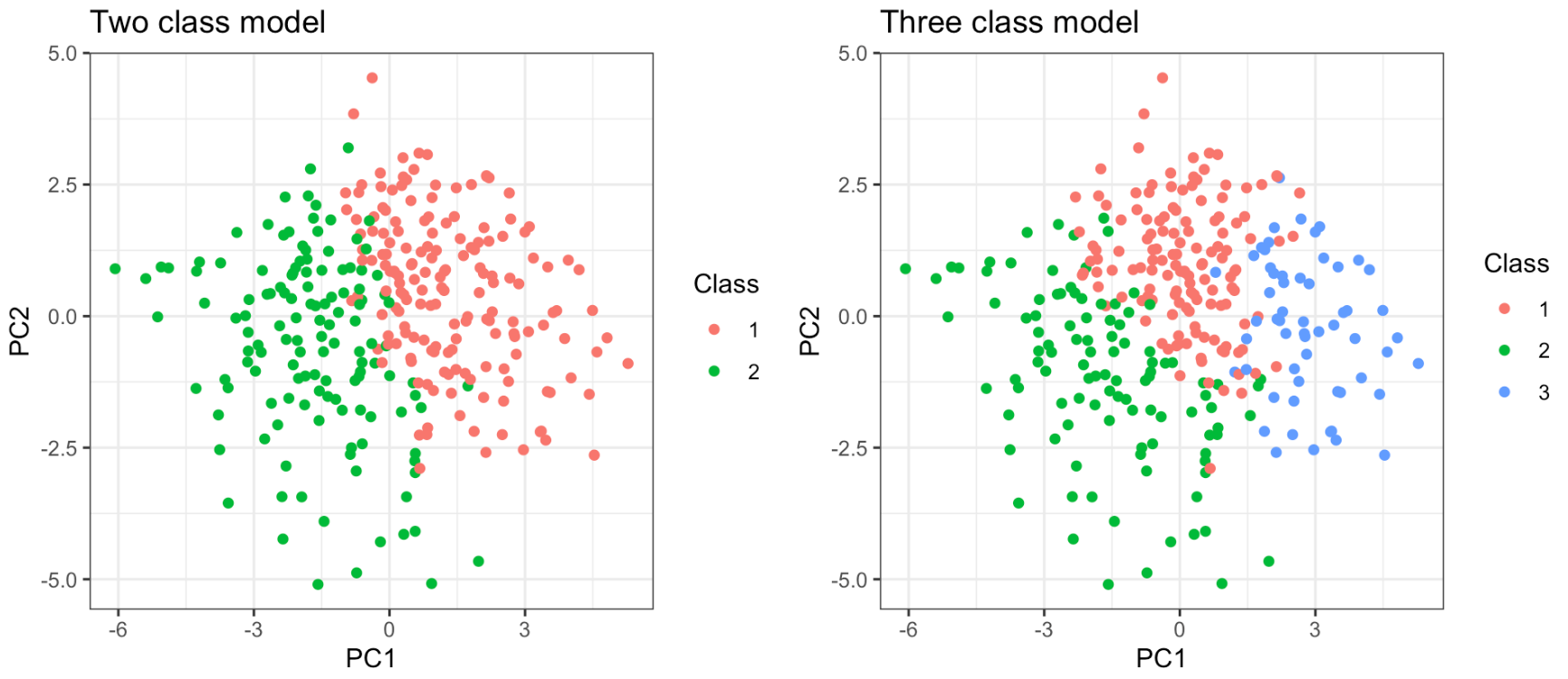


* Description: PC1 and PC2 represent the two first principal components of all variables used in the LCA.

**ESM-table 2. Pearson correlations of class defining variables with absolute correlations over 0.5**

|  | sTM | HBP | MMP8 | PRTN3 | Ela | Creatinine | Base Excess | pH |
| --- | --- | --- | --- | --- | --- | --- | --- | --- |
| HBP | 0.26 |  |  |  |  |  |  |  |
| MMP8 | 0.10 | 0.46 |  |  |  |  |  |  |
| PRTN3 | 0.34 | **0.54** | **0.53** |  |  |  |  |  |
| Ela | 0.24 | **0.53** | 0.45 | **0.71** |  |  |  |  |
| Creatinine | **0.50** | 0.06 | 0.07 | 0.10 | 0.07 |  |  |  |
| Base Excess | -0.17 | -0.21 | -0.08 | -0.04 | -0.10 | -0.40 |  |  |
| pH | -0.06 | -0.08 | 0.08 | 0.00 | 0.01 | -0.26 | **0.68** |  |
| Lactate | 0.02 | 0.32 | 0.15 | 0.09 | 0.19 | 0.06 | **-0.51** | -0.35 |

* Description: The values are Pearson’s correlations between the variables. All class defining variables that had at least one correlation of above in absolute value 0.5 with another class defining variable are included. Correlations over 0.5 are presented as bolded.

**Logistic regressions**

We included partly overlapping covariates in order to control for the effect of disease severity. For example, APACHE II, SOFA score, and KDIGO AKI stage that are all related of organ dysfunction. However, multicollinearity was not a problem here for two reasons. First, multicollinearity only matters if the exposure of interest (class membership) is collinear with other covariates, since it only affects the estimation of coefficients for the variables that are multicollinear with each other. Second, for both 90-day mortality and short-term renal recovery, the generalized variance inflation factors (GVIF) for all predictors were below a conservative threshold of GVIF^(1/(2^*^df^*^))^ < 2 (maximum GVIF^(1/(2^*^df^*^))^ was at 1.18).

**Three class model associations with outcomes**

Repeating the logistic regressions presented in Table 2. for the three-class model, using class 1 as reference, membership in class 2 is significantly associated with reduced 90-day mortality with OR 0.27 (p<0.001) and with increased renal recovery with OR 2.26 (p=0.012). Membership in class 3 is not associated with mortality with OR 1.16 (p=0.715) and is associated with reduced renal recovery with OR 0.31 (p=0.004).

**ESM-Figure 2.** Heatmap of class assignments for the admission model across 31 imputations.

| **301 patients** | 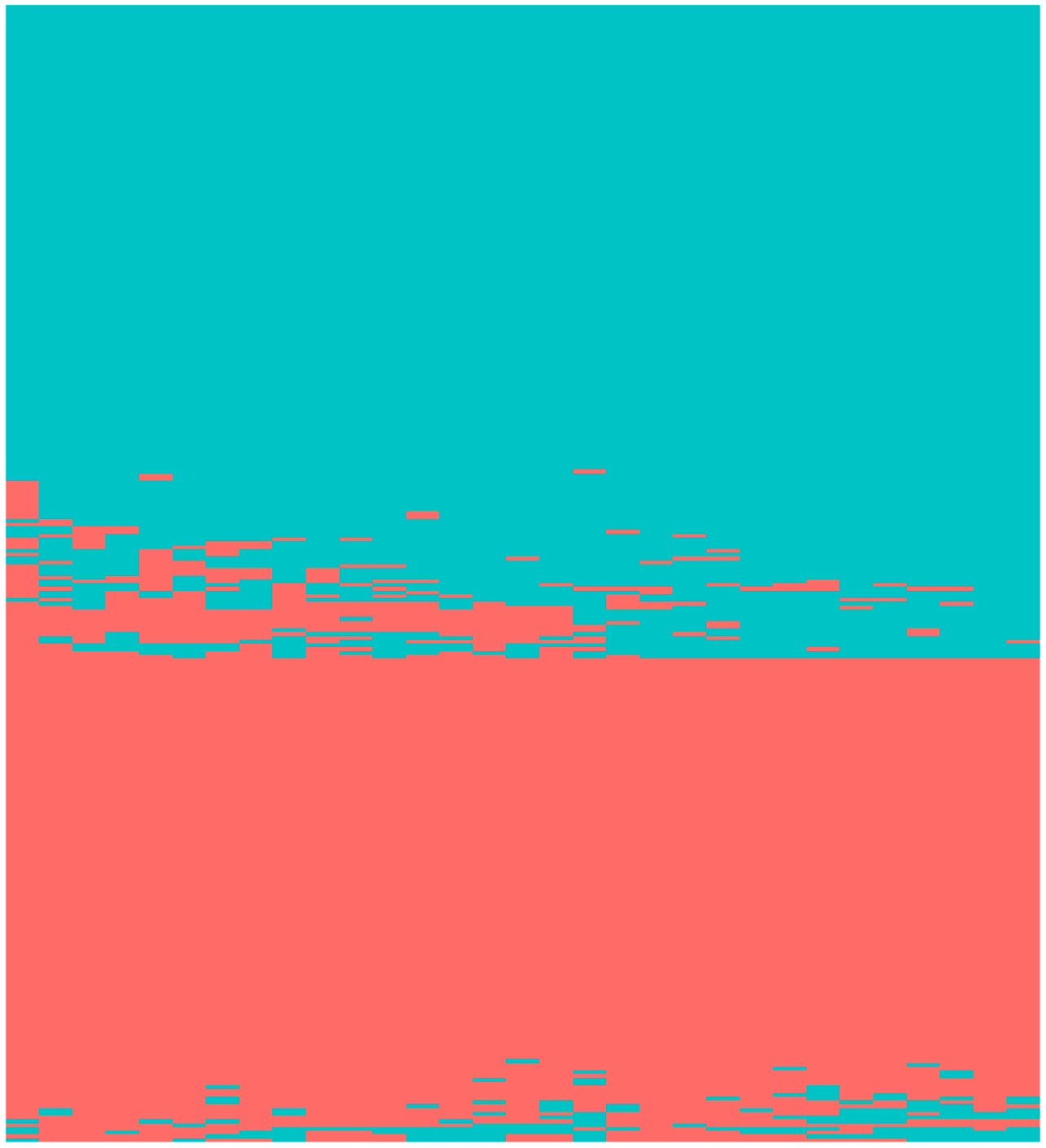 |
| --- | --- |
|  | **31 different imputed datasets** |

* Description: Heatmap contains each patient as a row of rectangles, and each column corresponds to a different, multiply-imputed datasets. The rectangles are coloured by the class of each patient in each multiply imputed dataset, teal (blue-green) denotes class 1 and red denotes class 2.

**ESM-table 3.** List of variables included in multiple imputation and percentages of missing data

| Variable name | % missing | Variable name | % missing |
| --- | --- | --- | --- |
| Syndecan-1 (SDC1) | 8.5 | Diabetes mellitus | 0.0 |
| Angiotensin 2 (ANG-2) | 6.7 | Body mass index | 0.3 |
| Interleukin 6 (IL 6) | 13.2 | Age | 0.0 |
| Soluble thrombomodulin (sTM) | 5.5 | Sex | 0.0 |
| Vascular Adhesion Protein 1 (VAP 1) | 5.5 | Day 1 urine output | 0.5 |
| Heparin Binding Protein (HBP) | 10.4 | Pneumonia on admission | 0.0 |
| CD73 | 31.9 | Day 1 highest WBC count | 1.5 |
| Fibroblast growth factor 13 (FGF13) | 14.8 | Day 1 lowest sodium | 3.7 |
| Olfactomedin 4 (OLFM4) | 12.0 | Day 1 lowest hematocrit | 31.5 |
| Matrix metalloproteinase 8 (MMP8) | 12.2 | Day 1 lowest platelets | 0.8 |
| Proteinase 3 (PRTN3) | 8.6 | Day 1 lowest base excess | 4.2 |
| Neutrophil elastase 2 (ELA2) | 8.6 | Day 1 highest creatinine | 1.8 |
| Study site | 0.0 | Day 1 vasopressor load | 28.9 |
| COPD | 1.0 | Day 1 mechanical ventilation | 0.0 |
| Hypertension | 0.5 | Operative admission | 0.2 |
| Atherosclerosis | 1.5 | Urinary tract infection on admission | 0.0 |
| Heart failure | 0.8 | Liver failure | 0.8 |
| Chronic kidney disease | 0.7 | Survivor status on 90 days | 0.0 |
| Rheumatoid disease | 1.0 | 5-day renal recovery | 0.0 |
| Diuretic use | 1.3 | Day 1 KDIGO AKI stage | 0.0 |
| Non-steroid immunosuppressant use | 1.0 | Admission PF ratio | 7.6 |
| Corticosteroid use | 0.7 | Admission noradrenaline rate | 0.0 |
| Warfarin use | 1.1 | 72h fluid balance | 21.5 |
| Pre ICU hypotension | 1.0 | Mechanical ventilation on admission | 0.0 |
| Pre ICU CPR | 0.2 | Admission mean arterial pressure | 6.5 |
| Pre ICU hypovolemia | 0.8 | Admission white blood cell count | 4.1 |
| ICU length of stay | 0.3 | Admission hematocrit | 1.6 |
| KDIGO AKI stage | 0.0 | Admission platelet count | 1.1 |
| Renal replacement therapy | 0.0 | Admission CRP | 2.8 |
| Day 1 noradrenaline, any use | 0.0 | Admission creatinine, highest | 2.0 |
| SOFA score | 0.0 | Admission base excess, lowest | 18.2 |
| SAPS II score | 0.0 | Admission pH, lowest | 17.9 |
| APACHE II score | 0.0 | Admission lactate, highest | 13.8 |
|  |  | Admission Creatinine | 0.3 |

**References**

1. Sterne JAC, White IR, Carlin JB, et al (2009) Multiple imputation for missing data in epidemiological and clinical research: potential and pitfalls. BMJ 338:b2393. https://doi.org/10.1136/bmj.b2393

2. Buuren S van, Groothuis-Oudshoorn K (2011) mice: Multivariate Imputation by Chained Equations in R. Journal of Statistical Software 45:1–67

3. Collins L, Lanza S (2009) Latent Class and Latent Transition Analysis: With Applications in the Social, Behavioral, and Health Sciences. Wiley

4. Iovleff S (2018) MixAll: Clustering and Classification using Model-Based Mixture Models

**ESM-table 4**. All included variables for clustering

| Admission model variable | Alternative in the 24h model |
| --- | --- |
| 1. Age |  |
| 2. Sex |  |
| 3. Body mass index |  |
| 4. Diabetes mellitus |  |
| 5. Liver failure |  |
| 6. Operative admission |  |
| Clinical variables | |
| 7. Mechanical ventilation on admission | Day 1 mechanical ventilation |
| 8. Pneumonia on admission |  |
| 9. Urinary tract infection on admission |  |
| 10. Mean arterial pressure, median of first 5 minutes |  |
| Biochemical variables | |
| 11. Platelet count* | Day 1 lowest platelets |
| 12. Creatinine, highest* | Day 1 highest creatinine |
| 13. pH, lowest* |  |
| 14. C - reactive protein |  |
| 15. Hematocrit* | Day 1 lowest hematocrit |
| 16. White blood cell count* | Day 1 highest white blood cell count |
| 17. Highest lactate* |  |
| 18. Base excess, lowest* | Day 1 lowest base excess |
| 19. Interleukin 6 (IL 6) |  |
| 20. Soluble thrombomodulin (sTM) |  |
| 21. Heparin Binding Protein (HBP) |  |
| 22. Vascular Adhesion Protein 1 (VAP 1) |  |
| 23. CD73 |  |
| 24. Neutrophil elastase 2 (ELA2) |  |
| 25. Matrix metalloproteinase 8 (MMP8) |  |
| 26. Fibroblast growth factor 13 (FGF13) |  |
| 27. Angiotensin 2 (ANG-2) |  |
| 28. Syndecan-1 (SDC1) |  |
| 29. Olfactomedin 4 (OLFM4) |  |
| 30. Proteinase 3 (PRTN 3) |  |

* Description: Admission model variables marked with * were measured during the interval from 24h prior to admission to 2h after admission.

**ESM-table 5**. Comparison of baseline characteristics between included and excluded patients

|  | Included (n=301) | Excluded (n=53) | P-value |
| --- | --- | --- | --- |
| Age, years | 65 (16) | 65 (15) | 0.88 |
| Sex, male | 28 (53%) | 185 (61%) | 0.24 |
| Body Mass Index, kg/m^2^ | 28.8 (8.7) | 28.6 (6.9) | 0.86 |
| Operative admission | 7 (13.2%) | 79 (26.3%) | 0.040 |
| Diabetes mellitus | 17 (32.1%) | 88 (29.2%) | 0.68 |
| Chronic liver failure | 6 (11.3%) | 10 (3.3%) | 0.010 |
| Chronic kidney disease | 8 (15.1%) | 24 (8.1%) | 0.10 |

**ESM-table 6**. Baseline of patients in class of 24 hour variable model

|  | Subphenotype 1  (n=130) | Subphenotype 2 (n=171) | P-value |
| --- | --- | --- | --- |
| Age, years, (SD) | 64 (15) | 65 (15) | 0.32 |
| Sex, male | 86 (66%) | 99 (58%) | 0.14 |
| BMI, kg/m^2^, (SD) | 29.6 (7.9) | 27.9 (6.0) | 0.027 |
| Operative admission | 51 (39.2%) | 77 (45.3%) | 0.035 |
| Diabetes mellitus | 44 (33.8%) | 44 (25.7%) | 0.13 |
| Chronic liver failure | 5 (3.9%) | 5 (3.0%) | 0.67 |
| Chronic kidney disease | 15 (11.7%) | 9 (5.3%) | 0.045 |
| Clinical variables on admission |  |  |  |
| Urinary tract infection | 10 (7.7%) | 22 (12.9%) | 0.15 |
| Pneumonia | 27 (20.8%) | 24 (14.0%) | 0.12 |
| Mechanical ventilation | 68 (52.3%) | 82 (48.0%) | 0.45 |
| Vasopressors, any | 60 (46.2%) | 111 (64.9%) | 0.001 |
| KDIGO AKI stage, Stage 1 | 62 (47.7%) | 68 (39.8%) | 0.34 |
| Stage 2 | 22 (16.9%) | 26 (15.2%) |  |
| Stage 3 | 34 (26.2%) | 61 (35.7%) |  |
| SOFA on admission, (SD) | 6.0 (2.7) | 6.4 (2.5) | 0.16 |
| APACHE score, (SD) | 26.8 (9.2) | 28.2 (8.6) | 0.18 |
| Mean arterial pressure, mmHg, (SD) | 77 (21) | 74 (22) | 0.16 |
| Biochemical variables on admission | |  |  |
| Leukocyte count, × 10^9^/L, (SD) | 12 (6) | 14 (10) | 0.046 |
| Platelet count, × 10^9^/L, (SD) | 234 (126) | 201 (142) | 0.039 |
| Hematocrit, (SD) | 0.4 (0.1) | 0.3 (0.1) | 0.48 |
| CRP, nmol/L, (SD) | 125.3 (112.9) | 231.1 (142.6) | <0.001 |
| pH, (SD) | 7.3 (0.2) | 7.3 (0.1) | 0.91 |
| Highest lactate, mmol/L, (SD) | 4.0 (4.5) | 4.5 (3.6) | 0.26 |
| Base excess, lowest, (SD) | -6.0 (9.2) | -8.4 (6.6) | 0.017 |
| Creatinine, µmol/L, (SD) | 213.8 (245.4) | 211.5 (167.2) | 0.92 |
| Treatment |  |  |  |
| Fluid balance at 72 hours, mL, (IQR) | 6820 (2145-10169) | 8124 (4202-13886) | 0.010 |
| Vasopressor load, μg/kg/min, (IQR) | 0.15 (0.04-0.44) | 0.28 (0.13-0.71) | 0.009 |
| Outcomes |  |  |  |
| Renal recovery | 85 (63.9%) | 78 (46.4%) | 0.009 |
| 90-day mortality | 39 (29.3%) | 68 (40.5%) | 0.045 |

* Abbreviations: BMI = Body Mass Index, KDIGO = Kidney Disease Improving Global Outcome, AKI = Acute Kidney Injury, SOFA = Sequential Organ Failure Assessment, APACHE = Acute Physiology, Age, Chronic Health Evaluation, CRP = C - reactive protein. Data are presented as numbers (percentages) or mean (SD) / median (IQR).

**ESM-table 7**. Comparison of admission and 24 hour model classification*

|  | Subphenotype 1  admission model | Subphenotype 2  admission model | Total |
| --- | --- | --- | --- |
| Subphenotype 1  24 hours model | 124 | 6 | **130** |
| Subphenotype 2  24 hours model | 9 | 162 | **171** |
| Total | **133** | **168** |  |

***** Description: comparison of classification based on admission and 24 hour model. From the 133 patients earlier classified as subphenotype 1, classification changed for nine patients to subphenotype 2 in the 24 hour model. Some of them were for example not mechanically ventilated or on vasopressors at admission, but did receive these during the first 24 hours. Conversely, from the 168 patients who were classified as being subphenotype 2 using variables on admission, six were reclassified to subphenotype 1 using the 24 hour model.

**ESM-figure 3**. Flowchart of patient inclusion


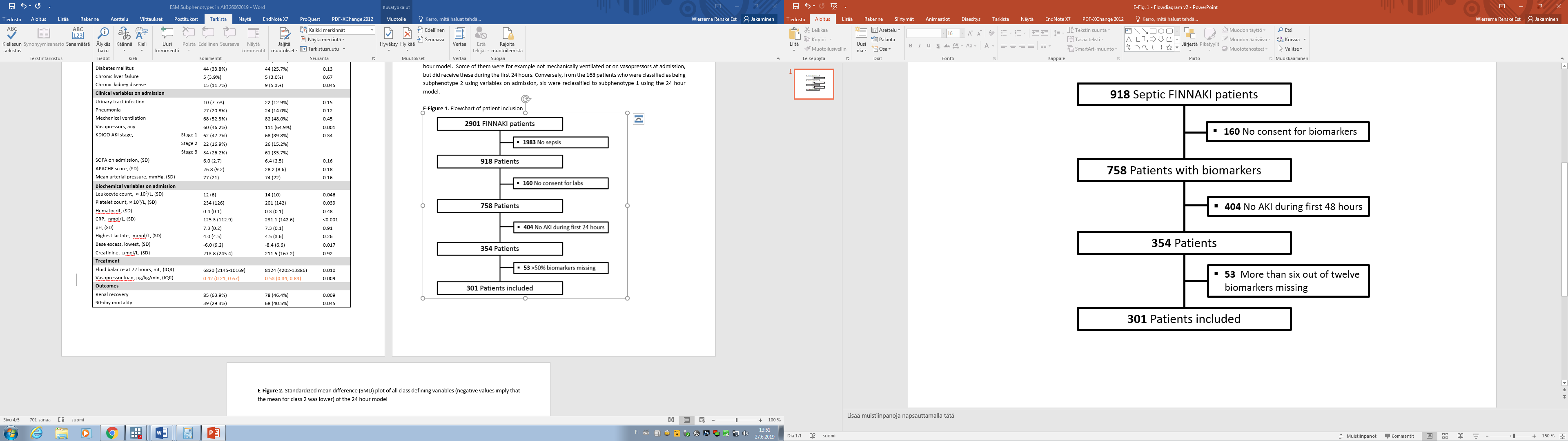


**ESM-figure 4.** Standardized mean difference (SMD) plot of all class defining variables (negative values imply that the mean for class 2 was lower) of the 24 hour model.


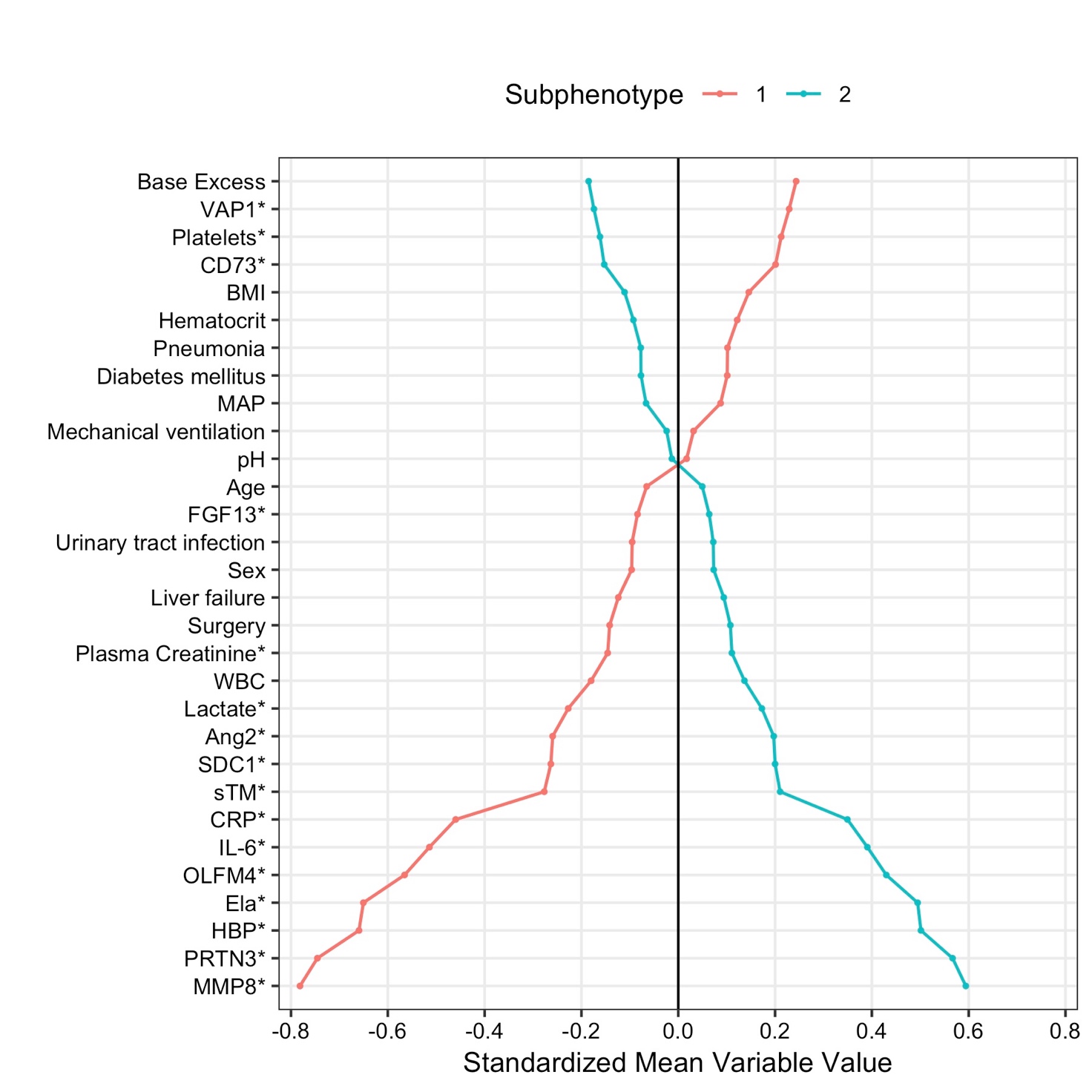


* Description: variables names with an * were plotted as either natural log or square root transformed. Every variable was standardized to a mean of 0 and SD of 1. Group means of standardized values are shown by class. The distance between the lines corresponds to the standardized mean difference between groups. Abbreviations: BMI = Body Mass Index, MAP = Mean Arterial Pressure, WBC = White Blood Cell Count, CRP = C- Reactive Protein, Ela = Neutrophil elastase 2, MMP8 = Matrix metalloproteinase 8, FGF13 = Fibroblast growth factor 13, OLFM4 = Olfactomedin 4, PRTN3 = Proteinase 3, sTM = Soluble thrombomodulin, SDC1 = Syndecan-1, VAP1 = Vascular Adhesion Protein 1, Ang2 = Angiotensin 2, IL-6 = Interleukin 6, HBP = Heparin Binding Protein.
